# Polydextrose Alleviates Adipose Tissue Inflammation and Modulates the Gut Microbiota in High-Fat Diet-Fed Mice

**DOI:** 10.3389/fphar.2021.795483

**Published:** 2022-02-02

**Authors:** Qiuyue Hu, Yixin Niu, Yanxia Yang, Qianyun Mao, Yao Lu, Hui Ran, Hongmei Zhang, Xiaoyong Li, Hongxia Gu, Qing Su

**Affiliations:** ^1^ Department of Endocrinology, Xinhua Hospital, Shanghai Jiao Tong University School of Medicine, Shanghai, China; ^2^ Department of Endocrinology, Xinhua Hospital Chongming Branch, Shanghai Jiao Tong University School of Medicine, Shanghai, China

**Keywords:** polydextrose, gut microbiota, adipose tissue inflammation, macrophage polarization, glucolipid metabolism

## Abstract

The soluble dietary fiber polydextrose (PDX) is a randomly linked glucose oligomer containing small amounts of sorbitol and citric acid and is widely used in the food industry. However, whether PDX can prevent and treat obesity in high-fat diet (HFD)-fed mice has not been directly investigated, and further studies are needed to better understand the complex interactions among PDX, adipose tissue inflammation and the gut microbiota. In the present study, PDX reduced body weight, fasting blood glucose (FBG), adipose tissue accumulation, adipocyte hypertrophy, serum total cholesterol (TC), low-density lipoprotein cholesterol (LDL-C) and high-density lipoprotein cholesterol (HDL-C) levels in HFD-fed mice. Moreover, PDX alleviated serum lipopolysaccharide (LPS) levels and macrophage infiltration in epididymal adipose tissue and resulted in macrophage polarization toward the M2 phenotype. Gut microbiota analysis revealed that PDX promoted the growth of beneficial microbes such as *Bacteroides*, *Parabacteroides*, *Alloprevotella*, *Muribaculum*, *Akkermansia*, *Ruminococcaceae_UCG-014* and *UBA1819* in obese mice, which were negatively correlated with subcutaneous fat, epididymal fat, body weight, FBG, serum TC, HDL-C, LDL-C and LPS levels. Our results indicates that PDX can prevent and treat obesity in HFD-fed mice, specifically in alleviating glucolipid metabolism disorders and adipose tissue inflammation, which may be mediated by modulating the structure of the gut microbiota. Therefore, PDX may become a promising nondrug therapy for obesity.

## Introduction

Obesity is a global health problem that is characterized by weight gain, fat accumulation, insulin resistance, chronic inflammation and gut microbiota dysbiosis (Canfora et al., 2019; Xu et al., 2021). Obesity can lead to a series of comorbidities, including type 2 diabetes mellitus, nonalcoholic fatty liver disease, cancer, cardiovascular diseases, hypertension, neurodegenerative diseases and sleep apnea (Blüher, 2019; Wilding et al., 2019). One study predicted that nearly 1 in 2 adults will have obesity and 1 in 4 adults will have severe obesity by 2030 in the United States (Ward et al., 2019). Obesity is associated with an increased risk of morbidity and mortality as well as a decrease in life expectancy (Martel et al., 2017; LeBlanc et al., 2018). Recently, many studies have indicated that individuals with obesity are associated with an increased risk of coronavirus disease 2019 (COVID-19) morbidity and mortality, and obese patients with COVID-19 infection should be treated more aggressively (Hamer et al., 2020; Popkin et al., 2020; Sanchis-Gomar et al., 2020; Stefan et al., 2020). Therefore, it is crucial and urgent for us to find effective therapies to prevent and treat obesity.

Dietary fiber is defined as carbohydrate polymers with three or more monomeric units that are neither digested nor absorbed in the small intestine. Dietary fiber is generally categorized into two classes: soluble dietary fiber such as inulin and β-glucan and insoluble dietary fiber such as cellulose. A large number of studies have indicated that dietary fiber intake is negatively correlated with chronic metabolic disorders and promotes health (Makki et al., 2018; Zhao and Zhang, 2018). Previous studies have revealed that obesity is often characterized by an increase in the proportion of *Firmicutes* and a decrease in *Bacteroides* (Ye et al., 2021; Zhai et al., 2021). Notably, dietary fiber can regulate the gut microbiota structure and contribute to fermentation products, in particular, short-chain fatty acids (SCFAs), which are considered important signaling molecules and beneficial for human health (Koh et al., 2016; Makki et al., 2018). Polydextrose (PDX) is a highly branched and randomly bonded glucose polymer that is considered a soluble dietary fiber. PDX is not hydrolyzed in the small intestine and fermented in the colon by endogenous microbiota (do Carmo et al., 2016). Several clinical trials have indicated that PDX can increase fecal bulk, soften stools and increase defecation frequency in healthy or constipated people (Hengst et al., 2009; Costabile et al., 2012). In addition, a galacto-oligosaccharide and PDX mixture can significantly reduce the incidence of viral respiratory tract infections in infants (Luoto et al., 2014; Ranucci et al., 2018). Moreover, PDX can decrease pH and increase SCFA production in the cecum of Wistar rats undergoing Billroth II partial gastrectomy (do Carmo et al., 2018). A previous study also revealed that PDX can modulate the gut microbiota and attenuate serum triglyceride and cholesterol levels in 14-days Western diet-fed mice (Raza et al., 2017).

Adipose tissue inflammation is considered a hallmark of obesity and is closely associated with the development of type 2 diabetes and cardiovascular disease (Kusminski et al., 2016; Oikonomou and Antoniades, 2019). Overnutrition can cause metabolic and structural changes in adipocytes, which initiate an inflammatory program and the subsequent recruitment of proinflammatory macrophages (Reilly and Saltiel, 2017). Moreover, emerging studies have demonstrated that metabolic inflammation is characterized by alterations in gut microbiota structure (Tilg et al., 2020; Chassaing et al., 2021; Shealy et al., 2021).

PDX has been widely used in the food industry, however, whether PDX can prevent and treat obesity in high-fat diet (HFD)-fed mice has not been directly investigated. We aim to investigate the effects of PDX on glucolipid metabolism disorders, adipose tissue inflammation and the gut microbiota in HFD-fed mice, as well as the complex interactions among them.

## Materials and Methods

### Animal Experiment Design

**FIGURE 1:**
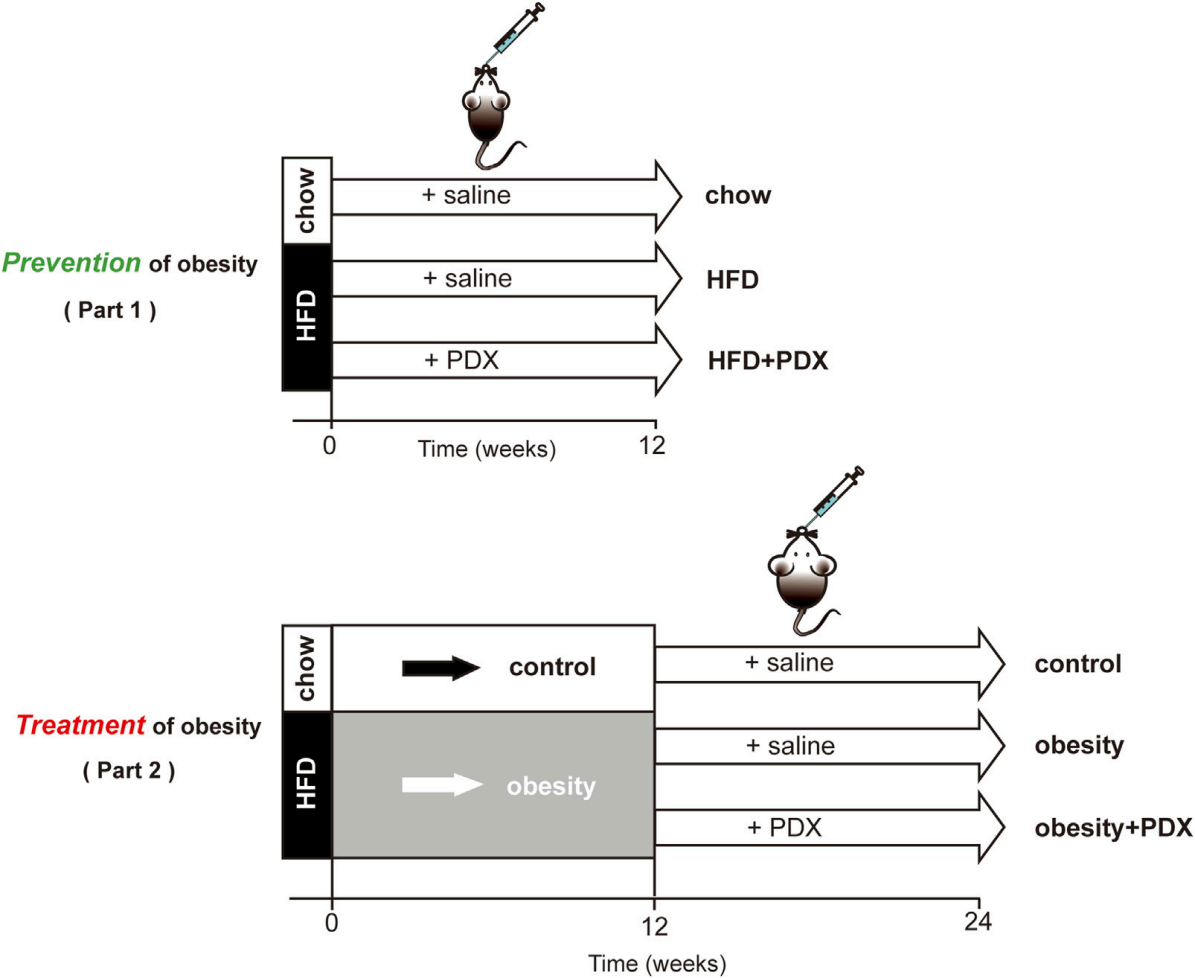
Schematic diagram of the two-part animal experiment design.

Part 1: Study on prevention of obesity. 4-week-old male C57BL/6 mice were purchased from Shanghai SLAC Laboratory Animal Co. Ltd. After 1 week of quarantine, the mice were randomly divided into three groups (12 mice per group): the chow, HFD and HFD + PDX groups. Mice in the chow group were fed a normal chow diet, while mice in the latter groups were fed a HFD (60% energy from fat; Research Diet, United States) supplemented or not with PDX (10 g/kg/day; Tate & Lyle Group, United Kingdom) for 12 weeks (Figure 1).

Part 2: Study on treatment of obesity. 4-week-old male C57BL/6 mice were randomly divided into two groups: the control and obesity groups. Mice in the obesity group were fed a HFD for 12 weeks to establish an obesity model. Then, these obese mice were randomly divided into two groups: the obesity and obesity + PDX groups (10 mice per group). The latter two groups were fed a HFD and left untreated or treated with PDX (10 g/kg/day) for another 12 weeks (Figure 1).

All of the mice were housed in the animal facility under a 12 h light/dark cycle and constant temperature (20–23°C). The mice had free access to sterile drinking water and food. Body weight was recorded once weekly, and fasting blood glucose (FBG) was measured every two weeks. At the end of the experiments, the mice were sacrificed under anesthesia. Serum samples and epididymal fat tissue were collected for further assays. Tissue samples were immediately submerged in liquid nitrogen and transferred to −80°C for storage. All procedures were performed in accordance with the National Institutes of Health Guidelines for the Care and Use of Animals and were approved by the Ethics Committee of Xinhua Hospital affiliated to Shanghai Jiao Tong University School of Medicine.

### Intraperitoneal Glucose Tolerance Test

The intraperitoneal glucose tolerance test (IPGTT) was conducted 1 week before the end of the experiment. The mice were fasted overnight for 16 h and then intraperitoneally injected with glucose (2 g/kg). Blood glucose was measured at 0, 15, 30, 60 and 120 min using a blood glucose meter (Bayer, Germany).

### Intraperitoneal Insulin Tolerance Test

The intraperitoneal insulin tolerance test (IPITT) was conducted at the end of the experiment. The mice were fasted for 6 h and then intraperitoneally injected with insulin (0.75 units/kg; Novo Nordisk, Denmark). Blood glucose was measured at 0, 15, 30, 60 and 120 min using a blood glucose meter (Bayer, Germany).

### Histology and Immunohistochemistry

Fresh epididymal adipose tissue samples from all parts of the mice were fixed in 4% paraformaldehyde for 24 h. Then, the tissues were embedded in paraffin wax and cut into 5 μm thick tissue slices. The slices were stained with hematoxylin and eosin (H&E) and observed with optical microscopy. ImageJ software was used to estimate adipocyte size. Additionally, the slices were deparaffinized and rehydrated. Then, the slices were placed in citric acid antigen repair buffer (pH 6.0), heated in a microwave oven for antigen repair, and incubated with 3% H_2_O_2_ for 25 min in the dark to block the activity of endogenous peroxidase. The slices were blocked in 3% BSA for 30 min, incubated with primary rabbit anti-mouse F4/80 antibody (Cell Signaling, MA, United States) at 4°C overnight, and then incubated with HRP-conjugated secondary antibody for 50 min at room temperature. Diaminobenzidine substrate was used to develop the color, and the slices were counterstained with hematoxylin. A Leica microscope was used for image acquisition, and ImageJ was used to calculate the percentage of F4/80-positive staining area per slice.

### Immunofluorescence

The slices were deparaffinized and rehydrated. Then, the slices were placed in citric acid antigen repair buffer (pH 9.0), heated in a microwave oven for antigen repair, and blocked in 3% BSA for 30 min. Next, the slices were incubated with primary F4/80 antibody (Cell Signaling, MA, United States) at 4°C overnight, followed by incubation with secondary antibody for 50 min at room temperature. Then, the slices were stained with DAPI and mounted.

### Biochemical Analyses

Blood samples were centrifuged at 3,000 × g for 10 min, and the supernatant was collected and stored at −80°C before the assay. The serum total triglyceride (TG), total cholesterol (TC), low-density lipoprotein cholesterol (LDL-C) and high-density lipoprotein cholesterol (HDL-C) levels of all the samples were detected with an automatic biochemical analyzer (Hitachi, Japan).

### Enzyme-Linked Immunosorbent Assay

Serum lipopolysaccharide (LPS), tumor necrosis factor-alpha (TNF-α), interleukin (IL)-1β, IL-6, IL-10 and fasting insulin (FINS) levels were measured according to the protocol of the corresponding ELISA kit (Westang, China).

### Quantitative Real-Time Polymerase Chain Reaction

Total RNA was extracted from epididymal adipose tissues using TRIzol reagent (Takara, Japan) according to the manufacturer’s instructions. Then, total RNA was reverse transcribed to complementary deoxyribonucleic acid (cDNA) with a reverse transcription reagent kit (Takara, Japan). The cDNA samples were subsequently amplified with SYBR Green PCR reagent (Takara, Japan) on an Applied Biosystems QuantStudio3 Real-Time PCR System (Thermo Fisher, MA, United States). The relative mRNA expression level of each gene was calculated by the comparative cycle threshold method (2^−ΔΔCt^), and the results were normalized to the expression level of the housekeeping gene β-actin. The primer sequences are provided in Table 1.

**TABLE 1:** Sequence of primers for quantitative real-time PCR

Gene	Species	Forward primer (5′→3′)	Reverse primer (5′→3′)
DGAT2	mouse	GCG​CTA​CTT​CCG​AGA​CTA​CTT	GGG​CCT​TAT​GCC​AGG​AAA​CT
SREBF1	mouse	TGA​CCC​GGC​TAT​TCC​GTG​A	CTG​GGC​TGA​GCA​ATA​CAG​TTC
Fasn	mouse	GGA​GGT​GGT​GAT​AGC​CGG​TAT	TGG​GTA​ATC​CAT​AGA​GCC​CAG
PPARα	mouse	TAC​TGC​CGT​TTT​CAC​AAG​TGC	AGG​TCG​TGT​TCA​CAG​GTA​AGA
CPT1α	mouse	TGG​CAT​CAT​CAC​TGG​TGT​GTT	GTC​TAG​GGT​CCG​ATT​GAT​CTT​TG
CD36	mouse	ATG​GGC​TGT​GAT​CGG​AAC​TG	TTT​GCC​ACG​TCA​TCT​GGG​TTT
HSL	mouse	CTC​ACA​GTT​ACC​ATC​TCA​CCT​C	GAT​TTT​GCC​AGG​CTG​TTG​AGT​A
LPL	mouse	GCC​GAG​AGC​GAG​AAC​ATT​CC	GCA​GTT​CTC​CGA​TGT​CCA​CC
Acsl3	mouse	CGG​AAA​TCA​TGG​ATC​GGA​TCT​A	GTG​GAG​TAC​TAC​ACC​CTT​TTG​A
TNF-α	mouse	ATG​TCT​CAG​CCT​CTT​CTC​ATT​C	GCT​TGT​CAC​TCG​AAT​TTT​GAG​A
IL-1β	mouse	TCG​CAG​CAG​CAC​ATC​AAC​AAG​AG	AGG​TCC​ACG​GGA​AAG​ACA​CAG​G
IL-6	mouse	CTG​CAA​GAG​ACT​TCC​ATC​CAG	AGT​GGT​ATA​GAC​AGG​TCT​GTT​GG
IL-10	mouse	TTC​TTT​CAA​ACA​AAG​GAC​CAG​C	GCA​ACC​CAA​GTA​ACC​CTT​AAA​G
MCP1	mouse	TTT​TTG​TCA​CCA​AGC​TCA​AGA​G	TTC​TGA​TCT​CAT​TTG​GTT​CCG​A
Nos2	mouse	AGG​CCA​CAT​CGG​ATT​TCA​CT	TCA​ATG​GCA​TGA​GGC​AGG​AG
CD11c	mouse	TCA​TCA​CTG​ATG​GGA​GAA​AAC​A	CCC​CAA​TTG​CAT​AAC​GAA​TGA​T
F4/80	mouse	TGT​CTG​CAT​GAT​CAT​CAC​GAT​A	CGT​GTC​CTT​GAG​TTT​AGA​GAC​T
Arg1	mouse	AGA​CCA​CAG​TCT​GGC​AGT​TGG	AGG​TTG​CCC​ATG​CAG​ATT​CCC
Fizz1	mouse	CAG​CTG​ATG​GTC​CCA​GTG​AAT	CAG​TGG​AGG​GAT​AGT​TAG​CTG​G
Mrc1	mouse	GGA​AGC​CCA​TTC​CGG​TAT​CT	CAT​CGC​TTG​CTG​AGG​GAA​TG
IL-4	mouse	GGA​CGC​CAT​GCA​CGG​AGA​TG	CGA​AGC​ACC​TTG​GAA​GCC​CTA​C
PPARγ	mouse	CCA​AGA​ATA​CCA​AAG​TGC​GAT​C	TCA​CAA​GCA​TGA​ACT​CCA​TAG​T
β-actin	mouse	GTG​ACG​TTG​ACA​TCC​GTA​AAG​A	GCC​GGA​CTC​ATC​GTA​CTC​C

### Western Blot Analysis

Proteins were extracted from epididymal adipose tissue using RIPA lysis buffer containing protease and phosphatase inhibitor cocktails (Beyotime, China), followed by quantification with a BCA protein quantitative analysis kit (Beyotime, China). Equal amounts of protein samples were separated on an SDS PAGE gel and transferred onto a polyvinylidene fluoride (PVDF) membrane (Millipore, United States). The membranes were then blocked with 5% skim milk powder and incubated with primary antibodies against β-actin, IκBα, p65 and p-p65 (Cell Signaling Technology, MA, United States) at 4°C overnight, followed by incubation with an HRP-conjugated secondary antibody (Beyotime, China) for 60 min at room temperature. Protein bands were visualized using Immobilon western chemiluminescent HRP substrate (Millipore, MA, United States) and then quantified with ImageJ software.

### Microbiota Analysis

In the last week of the Part 2 experiment, fresh feces were collected by anal stimulation or abdominal massage into sterile cryopreservation tubes, immediately submerged in liquid nitrogen and transferred to −80°C for storage. Microbial DNA was extracted from the fecal samples using a TIANamp Stool DNA kit (Qiagen, Germany) according to the manufacturer’s instructions. The V3-V4 region of the bacterial 16S ribosomal RNA gene was amplified by PCR using primers 343F (TACGGRAGGCAGCAG) and 798R (AGGGTATCTAATCCT). Amplicons were extracted from 2% agarose gels and purified using an AxyPrep DNA Gel Extraction Kit (Axygen Biosciences, CA, United States), followed by quantification using QuantiFluor™-ST (Promega, United States). Equal amounts of purified amplicons were sequenced on an Illumina MiSeq platform (Illumina, MA, United States) according to standard protocols. The raw sequencing data were obtained in FASTQ format. Ambiguous bases of paired-end reads were detected and trimmed using Trimmomatic software, and then paired-end reads were assembled using FLASH software. Clean reads were clustered to generate operational taxonomic units (OTUs) using Vsearch software with 97% identity. All representative reads were annotated and blasted against the Silva database using the RDP classifier (confidence threshold of 70%).

### Statistical Analysis

Data are shown as the mean ± standard error of the mean (SEM). Statistical analyses were performed using GraphPad Prism V.7.0. For parametric variables, the unpaired two-tailed Student’s t-test was used for comparisons between two groups, and one-way analysis of variance (ANOVA) followed by Bonferroni’s post hoc test was used for comparisons of three groups. For nonparametric variables, statistically significant differences were evaluated by the Wilcoxon rank-sum test or Kruskal–Wallis test with Dunn’s multiple comparisons test. To compare the body weight and FBG of three groups, two-way ANOVA followed by Bonferroni’s post hoc test was performed. The LEfSe method was used to assess the statistically significant difference in bacterial species. Spearman’s correlation analysis was performed to determine correlation coefficients between bacterial species and obesity traits. *p* < 0.05 was considered statistically significant.

## Results

### The Role of PDX in the Prevention of Obesity (Part 1)

**FIGURE 2:**
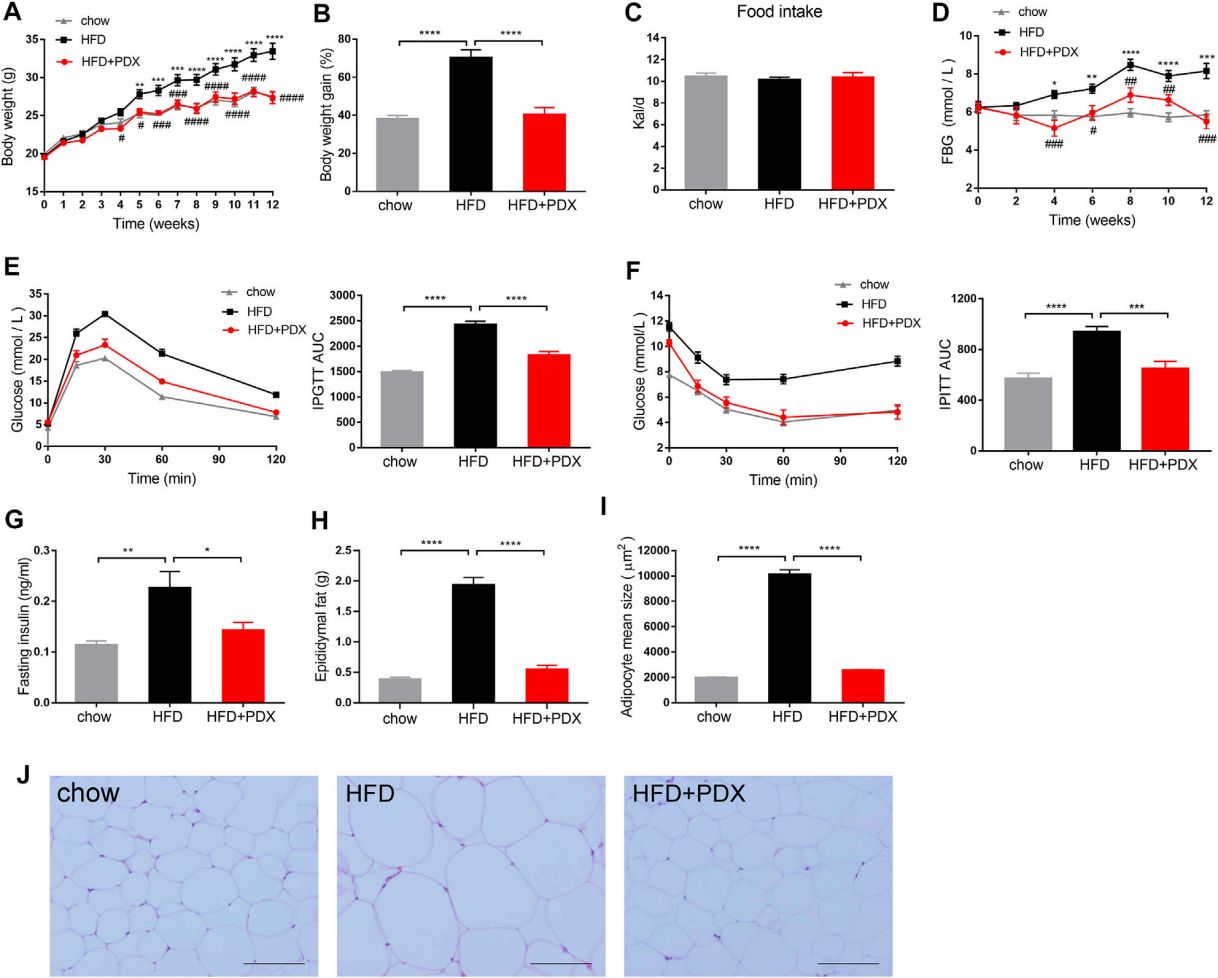
PDX prevented obesity-related traits in HFD-fed mice (Part 1). **(A)** Body weight. **(B)** Body weight gain at week 12. **(C)** Food intake measured as kilocalories per day. **(D)** FBG. **(E)** IPGTT and AUC measured during the IPGTT (mM⋅min). **(F)** IPITT and AUC measured during the IPITT (mM⋅min). **(G)** Fasting insulin. **(H)** Epididymal fat accumulation. **(I)** Adipocyte mean size. **(J)** Representative images of H&E-stained epididymal adipose tissues. Scale bar, 100 μm; magnification, x200. AUC, area under the curve. *n* = 12 per group. ^*^ *p* < 0.05; ^**^ *p* < 0.01; ^***^ *p* < 0.001; ^****^ *p* < 0.0001.

To investigate the effects of PDX on the prevention of obesity, mice were administered a HFD and PDX supplementation simultaneously for 12 weeks (Figure 1). The body weight and FBG of mice in the HFD group were significantly elevated compared with those of mice in the chow group (Figures 2A,B,D). In contrast, the HFD mice supplemented with PDX maintained a lower body weight and FBG than those of HFD group (Figures 2A,B,D). However, there was no significant difference in food intake among the three groups (Figure 2C). We also performed an IPGTT and an IPITT to study the effects of PDX on glucose tolerance and insulin sensitivity, respectively. The results indicated that the HFD decreased glucose tolerance and insulin sensitivity in mice; notably, PDX supplementation increased glucose tolerance and improved HFD-induced insulin resistance (Figures 2E,F). The FINS, epididymal fat accumulation and adipocyte size of the HFD group were increased significantly compared with those of the chow group. Additionally, PDX supplementation decreased all these metabolic parameters (Figures 2G–J).

**FIGURE 3:**
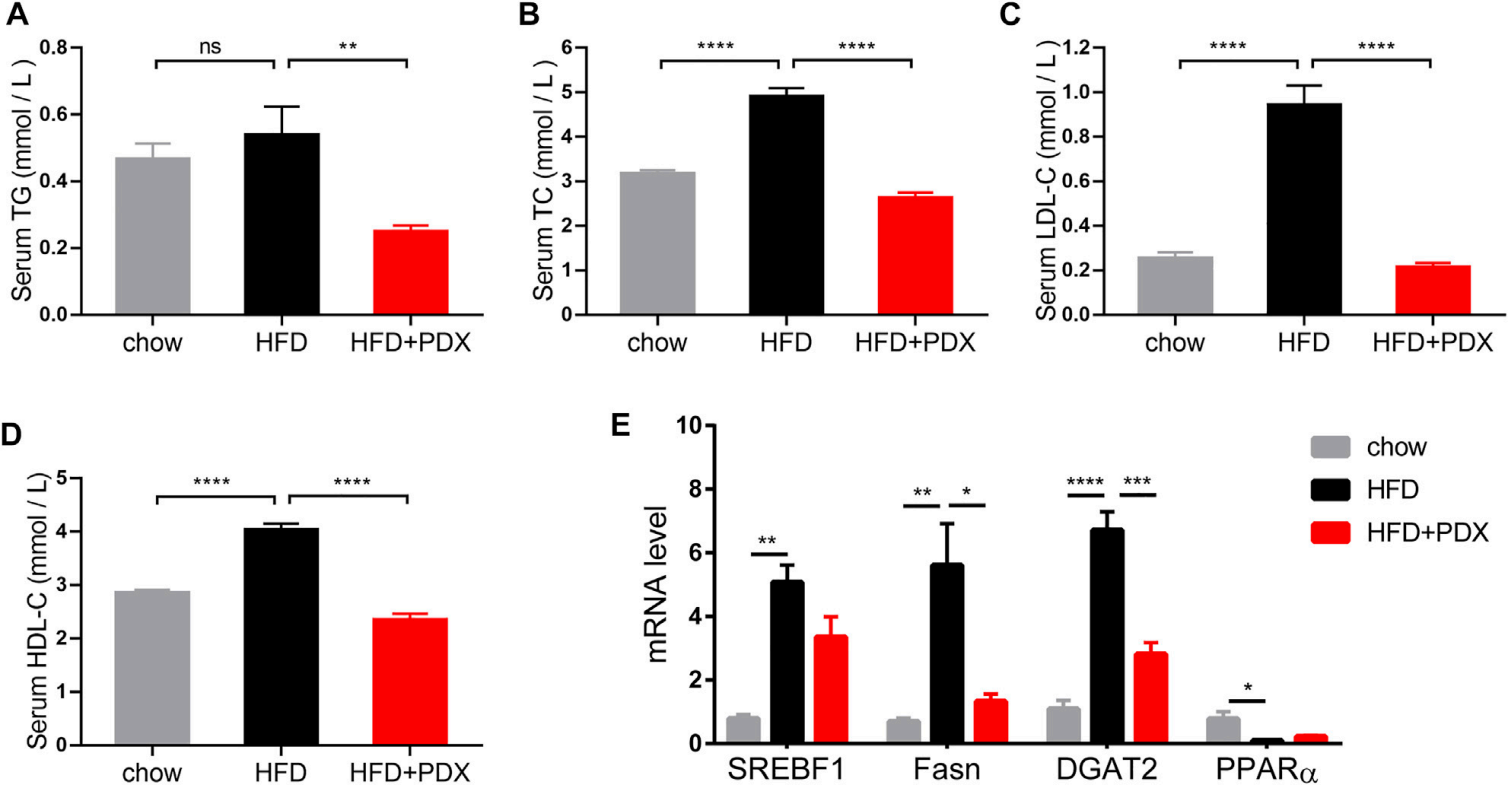
PDX prevented dyslipidemia in HFD-fed mice (Part 1). **(A)** Serum TG. **(B)** Serum TC. **(C)** Serum LDL-C. **(D)** Serum HDL-C. **(E)** qPCR analysis of genes involved in lipid metabolism SREBF1, Fasn, DGAT2 and PPARα in epididymal adipose tissue. *n* = 12 (A–D) or *n* = 4 **(E)** per group. ^*^ *p* < 0.05; ^**^ *p* < 0.01; ^***^ *p* < 0.001; ^****^ *p* < 0.0001; ns, not statistically significant.

We next explored whether PDX supplementation could prevent dyslipidemia in HFD-fed mice. Serum TC, LDL-C and HDL-C levels of the HFD group were increased compared with those of the chow group. In contrast, all serum lipid profiles as well as TG were reduced significantly in the HFD + PDX group (Figures 3A–D). To investigate whether PDX regulates the expression of genes involved in lipid metabolism, we examined genes involved in lipid transport, uptake, lipogenesis and lipolysis in adipose tissue. The results revealed that a HFD increased the expression of sterol regulatory element binding transcription factor 1 (SREBF1), fatty acid synthase (Fasn), and diacylglycerol acyltransferase 2 (DGAT2) in epididymal adipose tissue (Figure 3E). Notably, PDX supplementation reduced Fasn and DGAT2 levels in the epididymal adipose tissue of HFD-fed mice (Figure 3E).

**FIGURE 4:**
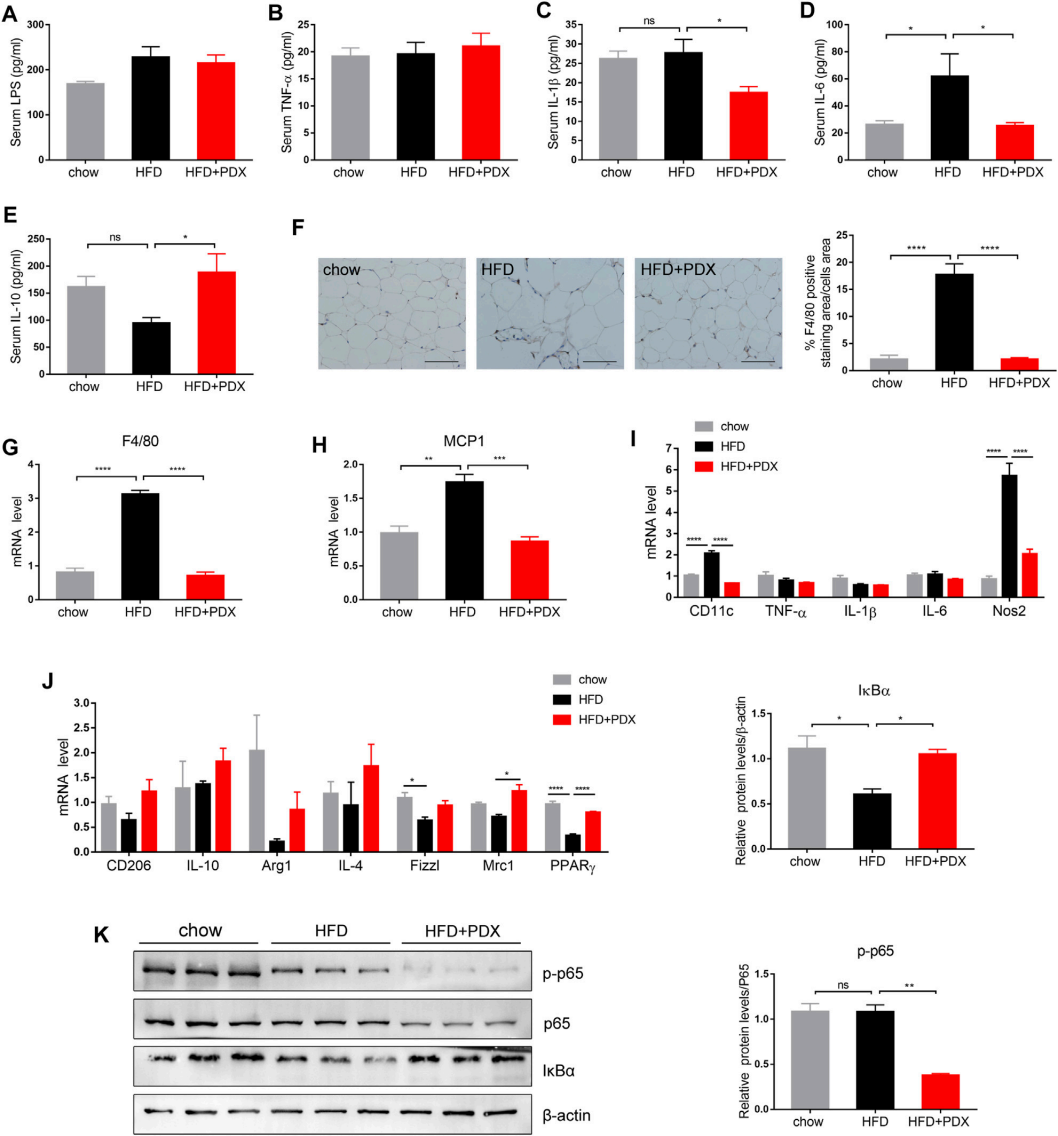
PDX maintained a lower inflammation level and promoted M2 macrophage polarization in the adipose tissue of HFD-fed mice (Part 1). **(A)** Serum LPS. **(B)** Serum TNF-α **(B)**. **(C)** Serum IL-1β. **(D)** Serum IL-6. **(E)** Serum IL-10. **(F)** Representative images of immunohistochemical staining of epididymal adipose tissue against the specific macrophage maker F4/80 and the quantification of F4/80-positive staining area. Scale bar, 100 μm; magnification, x200. **(G)** qPCR analysis of the mRNA levels of F4/80 in epididymal adipose tissue. **(H)** qPCR analysis of the mRNA levels of MCP1 in epididymal adipose tissue. **(I)** qPCR analysis of the mRNA levels of M1 macrophage maker CD11c, TNF-α, IL-1β, IL-6 and Nos2 in epididymal adipose tissue. **(J)** qPCR analysis of the mRNA levels of M2 macrophage maker CD206, IL-10, Arg1, IL-4, Fizz1, Mrc1 and PPARγ in epididymal adipose tissue. **(K)** Western blot analysis of the proteins expression involved in NF-κB signaling pathway in epididymal adipose tissue. *n* = 12 **(A–F)**, *n* = 4 **(G–J)** or *n* = 3 **(K)** per group. ^*^ *p* < 0.05; ^**^ *p* < 0.01; ^***^ *p* < 0.001; ^****^ *p* < 0.0001; ns, not statistically significant.

We further explored whether PDX could maintain a lower inflammation level in HFD-fed mice. The HFD group had a higher level of serum IL-6 compared with the chow group; however, PDX supplementation reduced serum IL-1β and IL-6 levels (Figures 4C,D). Moreover, PDX supplementation increased the serum level of the anti-inflammatory cytokine IL-10 (Figure 4E), and there was no significant difference in serum LPS and TNF-α levels among the three groups (Figures 4A,B).

To investigate whether PDX regulates macrophage infiltration and polarization, F4/80-positive macrophages were quantified by immunohistochemical staining in epididymal adipose tissue. The results revealed that a HFD largely increased the F4/80-positive staining area, and PDX supplementation significantly reversed macrophage infiltration (Figure 4F). In addition, the mRNA levels of F4/80 and monocyte chemoattractant protein-1 (MCP1) were increased in epididymal adipose tissue of the HFD group compared with that of the chow group and were reduced in that of the HFD + PDX group (Figures 4G,H). We next examined M1 and M2 macrophage markers in epididymal adipose tissue to further explore the regulatory effect of PDX on macrophage polarization. On the one hand, the results revealed that the mRNA levels of CD11c and nitric oxide synthase 2 (Nos2) were significantly increased in the HFD group compared with the chow group, and PDX supplementation reversed the change in the expression of CD11c and Nos2 (Figure 4I). On the other hand, the mRNA levels of Fizz1 and peroxisome proliferator-activated receptor γ (PPARγ) were decreased in the HFD group compared with the chow group, and PDX supplementation significantly increased mannose receptor 1 (Mrc1) and PPARγ levels in epididymal adipose tissue compared with a HFD alone (Figure 4J). Moreover, the results indicated that PDX supplementation increased IκBα (an inhibitor of the transcription factor NF-κB) levels and reduced p-p65 levels in epididymal adipose tissue of HFD-fed mice (Figure 4K).

### The Role of PDX in the Treatment of Obesity (Part 2)

**FIGURE 5:**
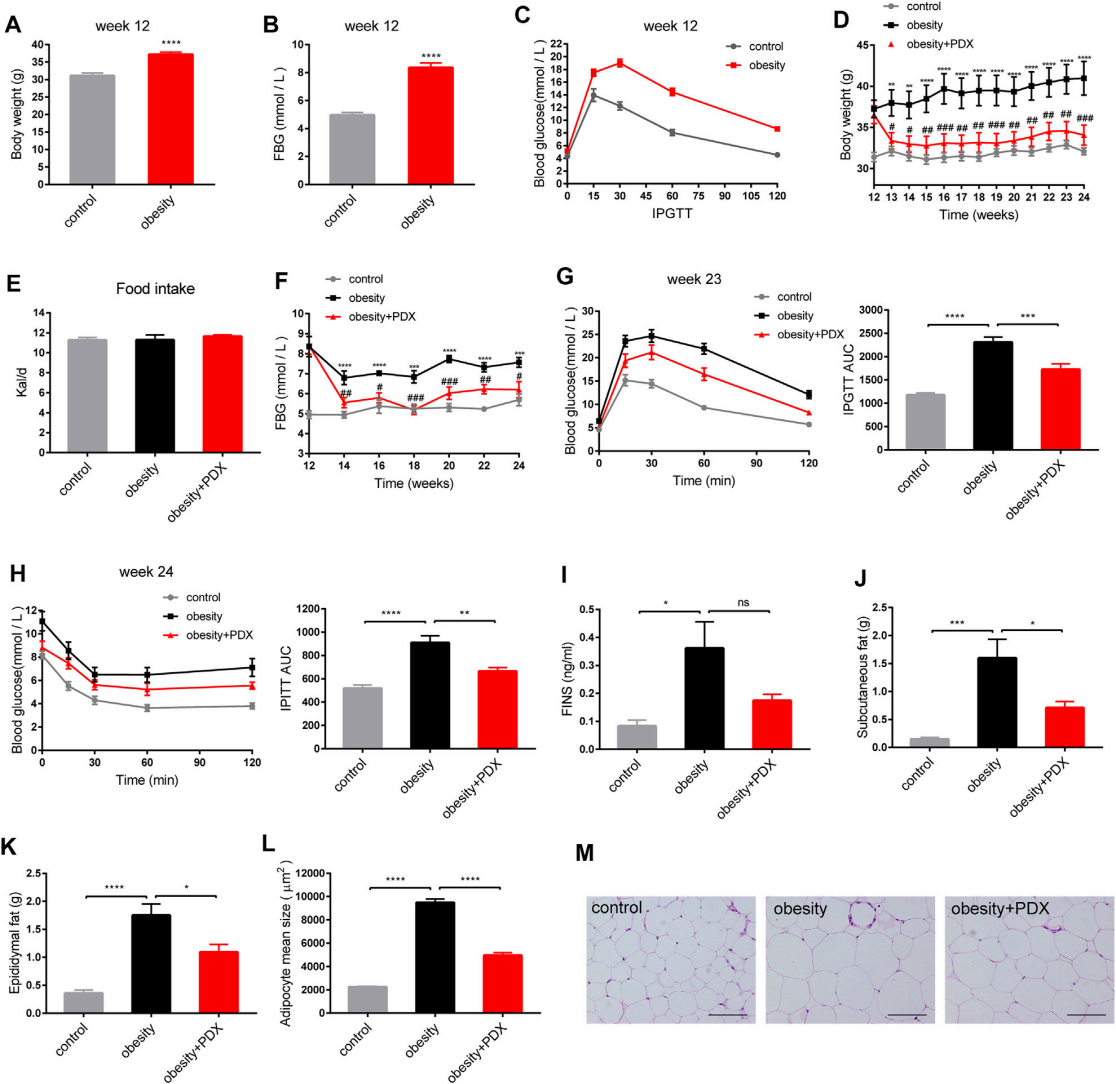
PDX alleviated obesity-related traits in HFD-induced obese mice (Part 2). **(A–C)** C57BL/6 mice were fed a HFD for 12 weeks to establish an obesity model. **(A)** Body weight at week 12. **(B)** FBG at week 12. **(C)** IPGTT at week 12. **(D–M)** The HFD-induced obese mice were then treated with PDX for another 12 weeks. **(D)** Body weight. **(E)** Food intake measured as kilocalories per day. **(F)** FBG. **(G)** IPGTT and AUC measured during the IPGTT at week 23 (mM⋅min). **(H)** IPITT and AUC measured during the IPITT at week 24 (mM⋅min). **(I)** FINS. **(J)** Subcutaneous fat accumulation. **(K)** Epididymal fat accumulation. **(L)** Adipocyte mean size. **(M)** Representative images of H&E-stained epididymal adipose tissues. Scale bar, 100 μm; magnification, x200. *n* = 10 per group. ^*^ *p* < 0.05; ^**^ *p* < 0.01; ^***^ *p* < 0.001; ^****^ *p* < 0.0001; ns, not statistically significant.

To investigate the effects of PDX on the treatment of obesity, an animal model of HFD-induced obesity was applied in our study (Figure 1). After 12 weeks of administering a HFD, the body weight and FBG of the obesity group were significantly increased compared with those of the control group (Figures 5A,B). Moreover, the IPGTT results showed that the HFD decreased glucose tolerance (Figure 5C). Then, obese mice were treated with PDX or sterile saline for another 12 weeks. After 12 weeks of PDX treatment, the body weight and FBG of the obesity + PDX group were significantly reduced compared with those of the obesity group (Figures 5D,F), and there was no significant difference in food intake among the groups (Figure 5E). The IPGTT and IPITT results indicated that PDX treatment increased glucose tolerance and improved obesity-associated insulin resistance (Figures 5G,H). Moreover, FINS, subcutaneous fat accumulation, epididymal fat accumulation and adipocyte size were all significantly decreased in the obesity + PDX group compared with obesity group (Figures 5I,K,L,M).

**FIGURE 6:**
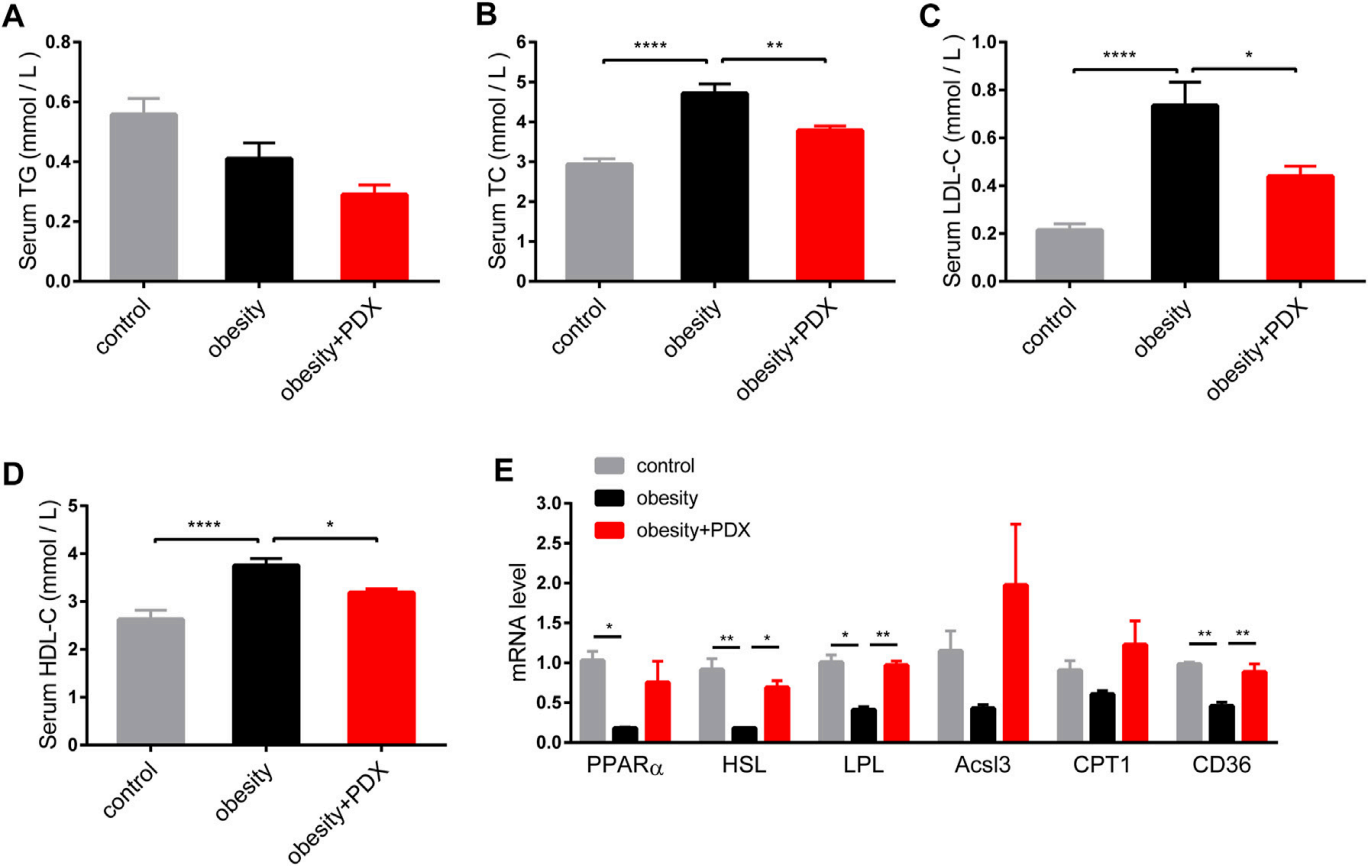
PDX improved dyslipidemia in HFD-induced obese mice (Part 2). **(A)** Serum TG. **(B)** Serum TC. **(C)** Serum LDL-C. **(D)** Serum HDL-C. **(E)** qPCR analysis of genes involved in lipid metabolism PPARα, HSL, LPL, Acsl3, CPT1 and CD36 in epididymal adipose tissue. *n* = 10 **(A–D)** or *n* = 4 **(E)** per group. ^*^ *p* < 0.05; ^**^ *p* < 0.01; ^***^ *p* < 0.001; ^****^ *p* < 0.0001.

As shown by the biochemical results, the serum TC, LDL-C and HDL-C levels in the obesity group were elevated compared with those in the control group, and PDX treatment reduced the levels of these lipid profiles (Figures 6B–D). In addition, the mRNA levels of peroxisome proliferator-activated receptor α (PPARα), hormone-sensitive triglyceride lipase (HSL), lipoprotein lipase (LPL) and CD36 in epididymal adipose tissue were reduced significantly in obese mice compared with mice in the control group (Figure 6E). Notably, PDX treatment increased HSL, LPL and CD36 levels in the epididymal adipose tissue of obese mice (Figure 6E).

**FIGURE 7:**
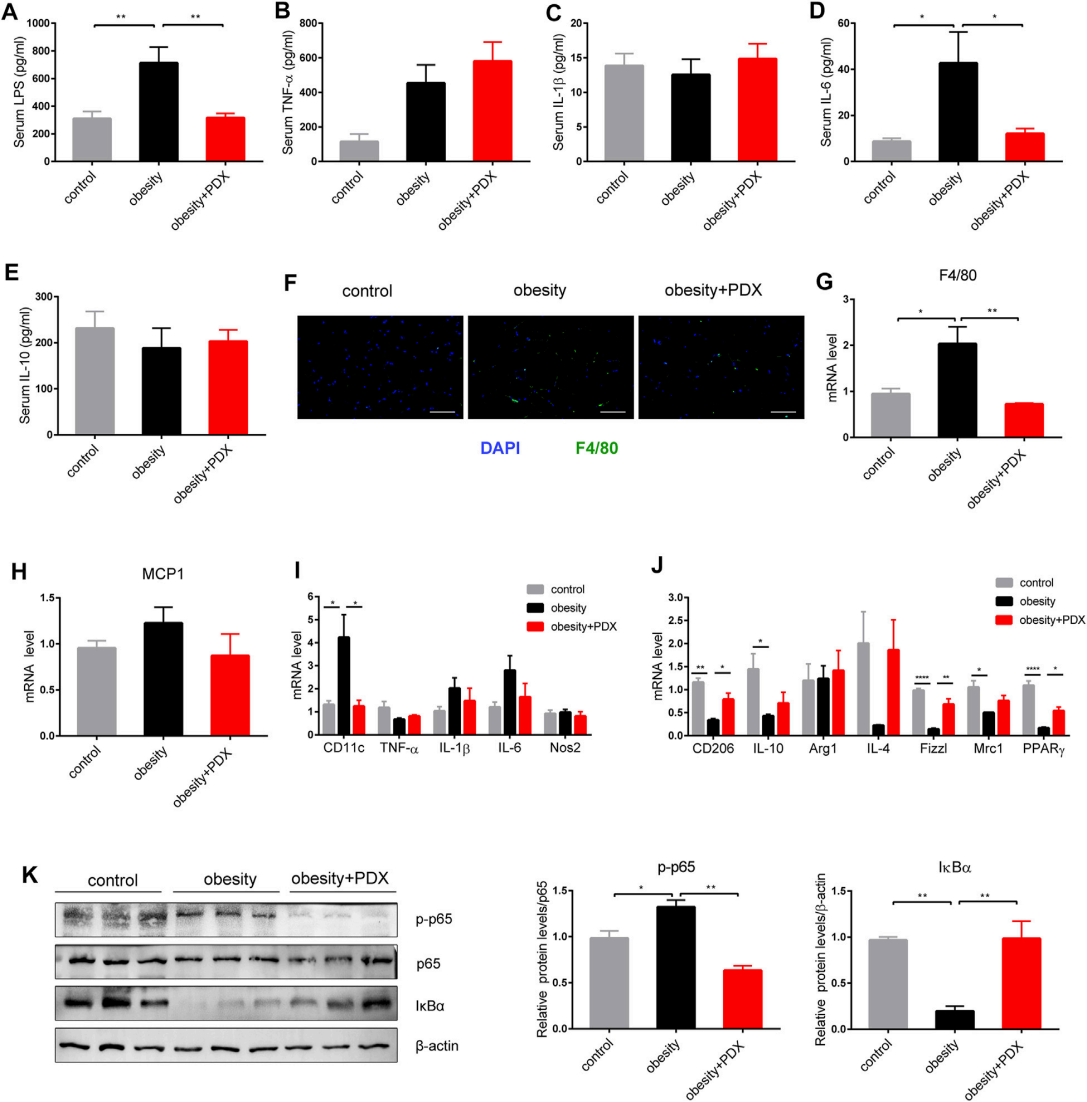
PDX alleviated inflammation levels and promoted macrophage polarization toward M2 phenotype in the epididymal adipose tissue of HFD-induced obese mice (Part 2). **(A)** Serum LPS. **(B)** Serum TNF-α **(B)**. **(C)** Serum IL-1β. **(D)** Serum IL-6. **(E)** Serum IL-10. **(F)** Representative images of immunofluorescence stain of epididymal adipose tissue against the specific macrophage maker F4/80. Scale bar, 100 μm; magnification, x200. **(G)** qPCR analysis of the mRNA levels of F4/80 in epididymal adipose tissue. **(H)** qPCR analysis of the mRNA levels of MCP1 in epididymal adipose tissue. **(I)** qPCR analysis of the mRNA levels of M1 macrophage maker CD11c, TNF-α, IL-1β, IL-6 and Nos2 in epididymal adipose tissue. **(J)** qPCR analysis of the mRNA levels of M2 macrophage maker CD206, IL-10, Arg1, IL-4, Fizz1, Mrc1 and PPARγ in epididymal adipose tissue. **(K)** Western blot analysis of the proteins expression involved in NF-κB signaling pathway in epididymal adipose tissue. *n* = 10 **(A–F)**, *n* = 4 **(G–J)** or *n* = 3 **(K)** per group. ^*^ *p* < 0.05; ^**^ *p* < 0.01; ^***^ *p* < 0.001; ^****^ *p* < 0.0001.

Obesity significantly increased the levels of serum LPS and IL-6, and PDX treatment reversed these levels of serum markers of inflammation (Figures 7A,D). There was no significant difference in serum TNF-α, IL-1β or IL-10 changes among the three groups (Figures 7B,C,E).

As shown by immunofluorescence staining, obesity largely increased the F4/80-positive staining area in epididymal adipose tissue, and PDX treatment significantly reduced macrophage infiltration (Figure 7F). Furthermore, F4/80 mRNA levels validated this result (Figure 7G). On the one hand, the CD11c mRNA level was significantly increased in epididymal adipose tissue of obesity group compared with the control group, and PDX treatment reversed the expression of CD11c (Figure 7I). On the other hand, the mRNA levels of CD206, IL-10, Fizz1, Mrc1 and PPARγ were reduced in obesity group, and PDX treatment significantly increased the levels of CD206, Fizz1 and PPARγ in epididymal adipose tissue (Figure 7J). Moreover, PDX treatment significantly increased the protein level of IκBα and reduced the level of p-p65 in the epididymal adipose tissue of obese mice (Figure 7K). The results indicated that PDX can inhibit NF-κB signaling in the epididymal adipose tissue of HFD-induced obese mice.

### PDX Modulates the Gut Microbiota Structure in HFD-Induced Obese Mice (Part 2)

**FIGURE 8:**
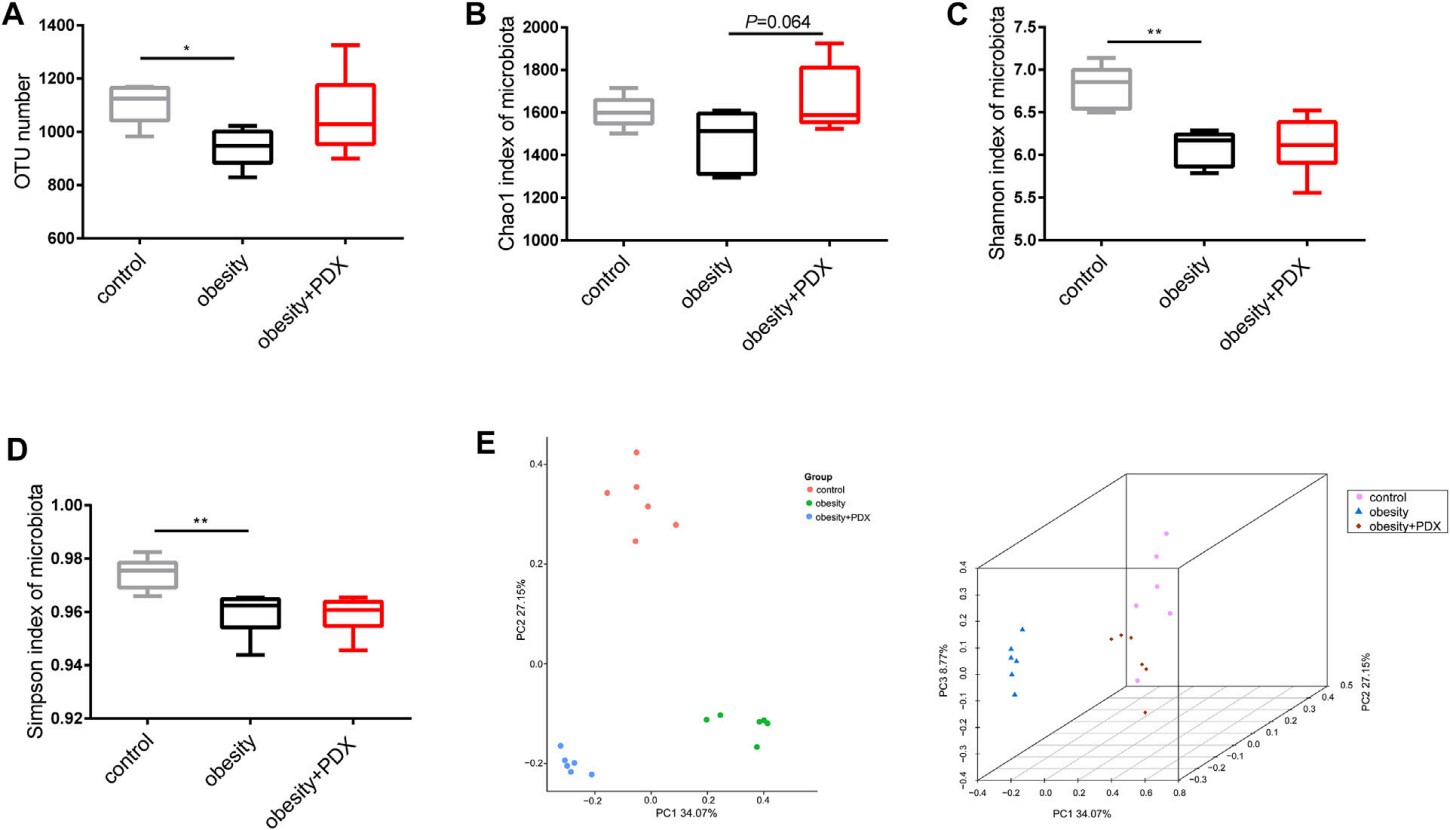
Alpha and beta diversity analysis of gut microbiota in HFD-induced obese mice (Part 2). **(A)** OTU number. **(B)** Chao1 index of microbiota. **(C)** Shannon index of microbiota. **(D)** Simpson index of microbiota. **(E)** 2D and 3D PCoA plot (Bray-Curtis index) of microbial community structure in obese mice. OTU, operational taxonomic unit; PCoA, principal coordinate analysis. *n* = 6 per group. ^*^ *p* < 0.05; ^**^ *p* < 0.01.

To explore the effect of PDX treatment on the gut microbiota in HFD-induced obese mice, we performed 16S rRNA sequencing of fecal samples. Compared with the control group, the obesity group had significantly fewer OTUs, and the PDX treatment group had slightly more OTUs (Figure 8A). However, the results revealed that there was no significant difference in the α diversity index (Chao1 index, Shannon index and Simpson index) between the obesity and obesity + PDX groups (Figures 8B–D). Principal coordinates analysis (PCoA) of the Bray-Curtis index for the gut microbiota showed that the control, obesity and obesity + PDX groups were clearly clustered into three separate groups (Figure 8E).

**FIGURE 9:**
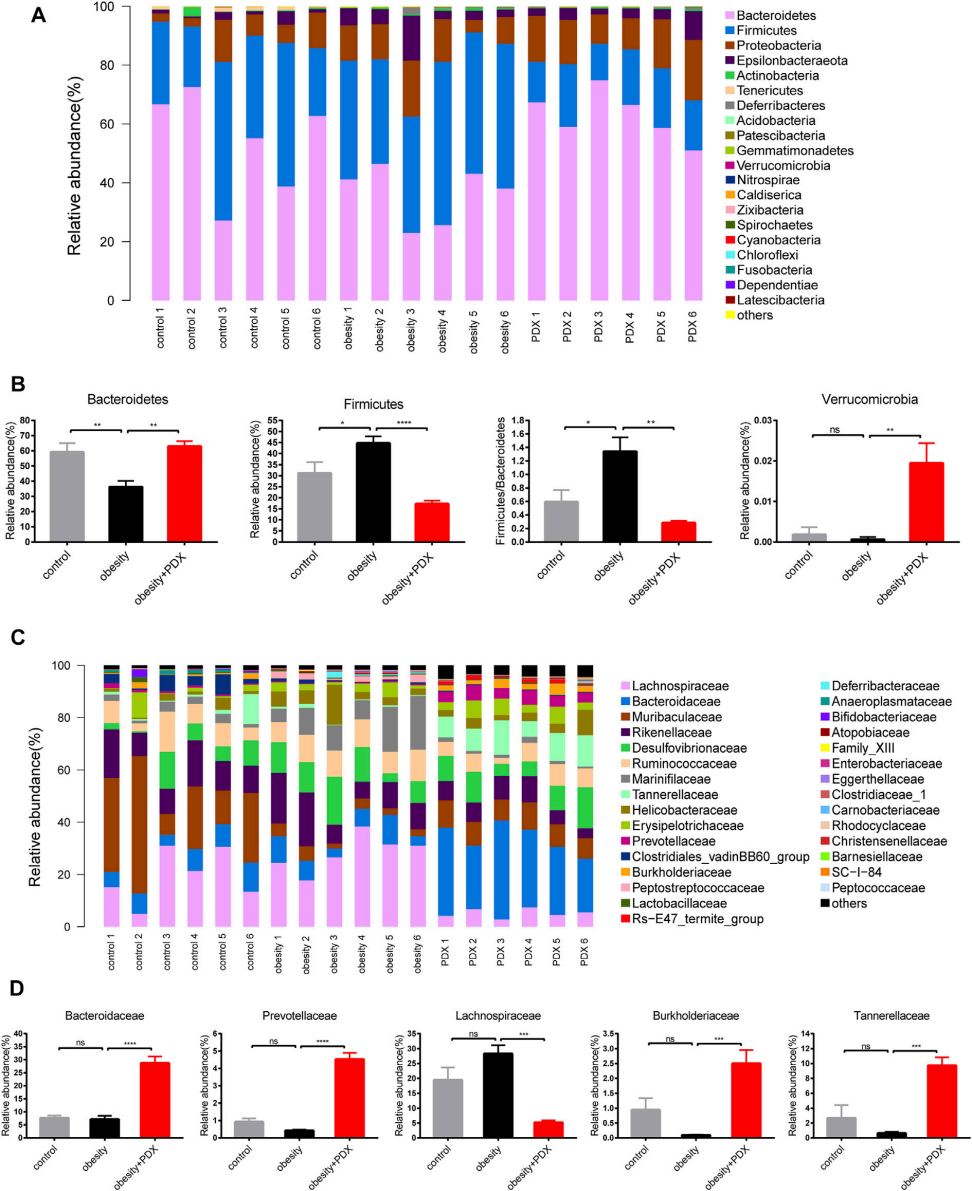
PDX modulated the composition of gut microbiota at the phylum and family level in HFD-induced obese mice (Part 2). **(A)** Relative abundance of microbial taxa at the phylum level. **(B)** Relative abundance of *Bacteroidetes*, *Firmicutes*, *Verrucomicrobia* and *Firmicutes/Bacteroidetes* ratio. **(C)** Relative abundance of microbial taxa at the family level. Top 30 abundances are shown. **(D)** Relative abundance of *Bacteroidaceae, Prevotellaceae, Lachnospiraceae, Burkholderiaceae and Tannerellaceae*. *n* = 6 per group. ^*^ *p* < 0.05; ^**^ *p* < 0.01; ^***^ *p* < 0.001; ^****^ *p* < 0.0001; ns, not statistically significant.

To further explore whether PDX treatment regulates gut microbiota structure in obese mice, the relative abundance of microbial taxa at different taxonomic levels was examined. At the phylum level, obesity reduced *Bacteroidetes* and increased *Firmicutes* abundance. However, *Bacteroidetes* and *Verrucomicrobia* were increased, and *Firmicutes* was reduced significantly under PDX treatment (Figures 9A,B). Correspondingly, the gut microbiota of obesity + PDX group showed a lower *Firmicutes/Bacteroidetes* ratio than that of obesity group (Figure 9B). At the family level, mice in obesity + PDX group had a higher abundance of *Bacteroidaceae*, *Prevotellaceae*, *Burkholderiaceae* and *Tannerellaceae* and a lower abundance of *Lachnospiraceae* than the mice in obesity group (Figures 9C,D).

**FIGURE 10:**
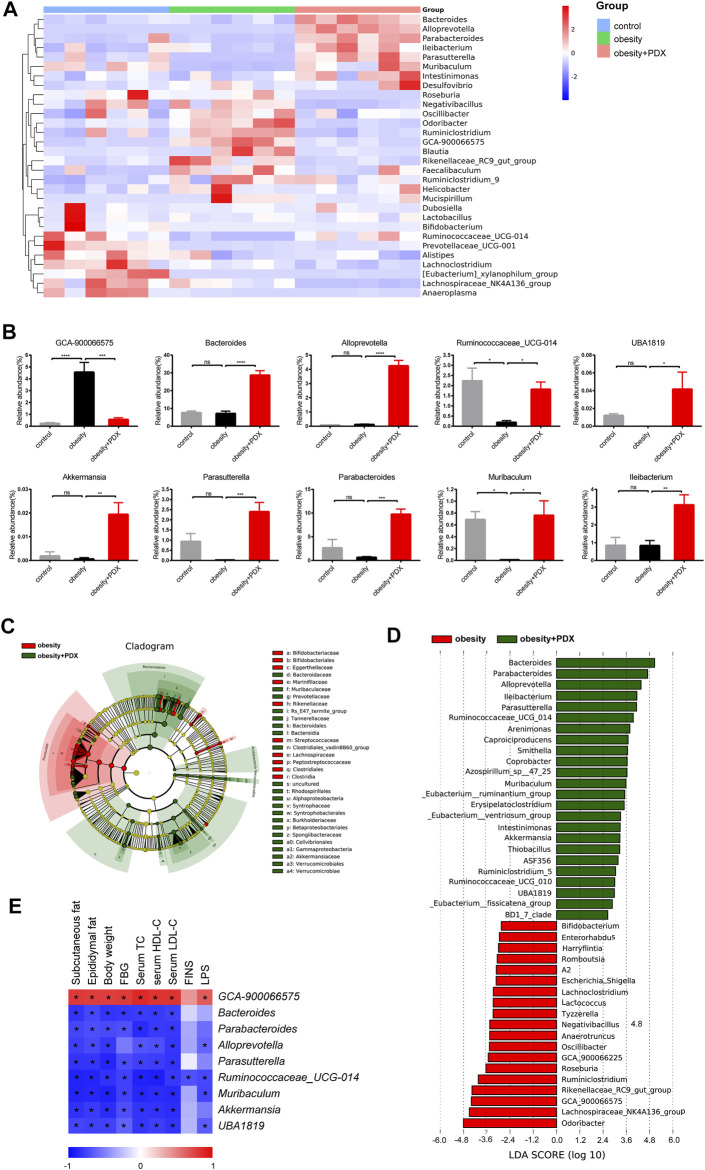
PDX modulated the composition of gut microbiota at the genus level in HFD-induced obese mice (Part 2). **(A)** Heatmap analysis using taxonomic abundances at the genus level. Top 30 abundances are shown. **(B)** Relative abundance of *GCA-900066575, Bacteroides*, *Alloprevotella*, *Ruminococcaceae* *_UCG-014*, *UBA 1819*, *Akkermansia*, *Parasutterella*, *Parabacteroides*, *Muribaculum* and *Ileibacterium*. **(C)** Cladogram derived from LEfSe analysis, showing the relationship between taxon. **(D)** Linear discriminant analysis (LDA) scores derived from LEfSe analysis, showing biomarker taxa at the genus level (LDA score >2). **(E)** Heatmap analysis of the Spearman correlations between 9 genera and 9 metabolic parameters. *n* = 6 per group. ^*^ *p* < 0.05; ^**^ *p* < 0.01; ^***^ *p* < 0.001; ^****^ *p* < 0.0001; ns, not statistically significant.

At the genus level, compared with the mice in the control group, obese mice had a higher abundance of *GCA-900066575* and a lower abundance of *Ruminococcaceae_UCG-014* and *Muribaculum* (Figures 10A,B). However, mice in obesity + PDX group had a higher abundance of *Bacteroides*, *Alloprevotella*, *Ruminococcaceae_UCG-014*, *UBA 1819*, *Akkermansia*, *Parasutterella*, *Parabacteroides*, *Muribaculum* and *Ileibacterium* and a lower abundance of *GCA-900066575* than the mice in obesity group (Figures 10A,B). To identify the biomarker taxa that differentiate obese mice and PDX-treated obese mice, we performed LEfSe analysis and selected genera based on an LDA score >2. The cladogram revealed that *Bacteroidetes* members played an important role in the effects of PDX, generating beneficial effects (Figure 10C). Correspondingly, we observed an overrepresentation of *Bacteroides*, *Parabacteroides*, *Alloprevotella*, *Ileibacterium*, *Parasutterella*, *Ruminococcaceae_UCG-014*, *Muribaculum*, *Akkermansia*, *ASF356*, *Ruminiclostridium_5* and *UBA1819* in the PDX treatment group compared with the obesity group; therefore, these taxa can be considered biomarker taxa (Figure 10D). Moreover, *GCA-900066575* was overrepresented in the obesity group compared with the obesity + PDX group (Figure 10D). To further analyze the association between these genera and metabolic phenotypes, we performed Spearman correlation analysis comparing 9 bacterial genera with 9 metabolic parameters. The results showed that *GCA-900066575* was significantly positively correlated with subcutaneous fat, epididymal fat, body weight, FBG, serum TC, HDL-C, LDL-C and LPS (Figure 10E). However, *Bacteroides*, *Parabacteroides*, *Alloprevotella*, *Parasutterella*, *Ruminococcaceae* *_UCG-014*, *Muribaculum*, *Akkermansia* and *UBA1819* were negatively correlated with these parameters (Figure 10E).

## Discussion

In the present study, we demonstrated that PDX exerted beneficial effects on the prevention and treatment of obesity. PDX alleviated glucolipid metabolism disorders and adipose tissue inflammation in HFD-fed mice. Moreover, we found that PDX treatment significantly modulated the gut microbiota structure in obese mice. Previous studies have indicated that gut microbiota dysbiosis is one of the reasons for obesity and related disorders, and fecal microbial transplantation (FMT) is considered a potential source of novel therapeutics (Napolitano and Covasa, 2020; He et al., 2021; Voland et al., 2021). FMT from the obesity-associated human gut microbiota to mice can induce vascular dysfunction and glucose intolerance, and FMT from lean donors to patients with obesity can promote metabolic benefits (Mocanu et al., 2021; Russo et al., 2021). Recent studies over the past decade have revealed that many environmental factors, including diet, antibiotic exposure, energy intake and exercise, can dramatically influence the gut microbiota (Du et al., 2021; Giordan et al., 2021; Shi et al., 2021).

Dietary fiber has been shown to improve obesity and is considered a potential therapy because it increases microbial fermentation of SCFAs (Schäfer et al., 2021; Thomson et al., 2021). A study indicated that PDX changes the gut microbiome, attenuates plasma TG and TC levels and regulates gene expression in the intestine of mice fed a Western diet for 14 days (Raza et al., 2017); however, we think the research period was somewhat short and not long enough to effectively improve metabolic disorders. Moreover, we performed a two-part study to certify the benefits of PDX on the prevention and treatment of obesity, which lasted for 12 or 24 weeks and focused on adipose tissue inflammation and gut microbiota modulation.

In our study, the PCoA results indicated that PDX treatment can modulate the gut microbiota in obese mice. PDX treatment increased the relative abundance of *Bacteroidetes* and *Verrucomicrobia* and decreased the relative abundance of *Firmicutes* and the *Firmicutes/Bacteroidetes* ratio in obese mice. On the one hand, the cladogram derived from LEfSe analysis revealed that *Bacteroidetes* members played an important role in the mediating the effects of PDX, generating beneficial effects; on the other hand, the genus LEfSe analysis indicated that *Bacteroides*, *Parabacteroides*, *Alloprevotella*, *Ileibacterium*, *Parasutterella*, *Ruminococcaceae* *_UCG-014*, *Muribaculum*, *Akkermansia*, *ASF356*, *Ruminiclostridium_5* and *UBA1819* were overrepresented in the obesity + PDX compared with the obesity group, and *GCA-900066575* was overrepresented in the obesity group. *Bacteroides* (family Bacteroidaceae), *Parabacteroides* (family Tannerellaceae), *Alloprevotella* (family Prevotellaceae) and *Muribaculum* (family Muribaculaceae) all belong to the phylum *Bacteroidetes*. *Bacteroidetes* members encode a proportionally higher number of carbohydrate-active enzymes (CAZymes such as glycoside hydrolases and polysaccharide lyases) than bacteria of other phyla, which enables the optimal use of dietary and host mucosal glycans (Lapébie et al., 2019). Similarly, a study indicated that curcumin alleviates HFD-induced hepatic steatosis and obesity via modulation of gut microbiota including *Akkermansia*, *Bacteroides* and *Parabacteroides* (Li et al., 2021) and these bacteria are all belong to SCFA-producing bacteria (Wu et al., 2021). *Akkermansia muciniphila* is widely considered a promising “next-generation beneficial microbe” for promoting beneficial effects on the metabolic disease, inflammatory bowel disease and tumour immunity, owing to various mechanisms including producing SCFAs, maintaining the integrity of gut barrier and reducing circulating LPS level (Yan et al., 2021; Yang et al., 2021; Zhang et al., 2021). Furthermore, *Parabacteroides goldsteinii* was reduced in HFD-fed mice, and oral treatment of the HFD-fed mice with live *Parabacteroides goldsteinii* reduced obesity (Wu et al., 2019). *Muribaculum* is reduced in mice with Crohn’s disease (Dobranowski et al., 2019). Moreover, Spearman correlation analysis proved that *Bacteroides*, *Parabacteroides*, *Alloprevotella*, *Parasutterella*, *Ruminococcaceae* *_UCG-014*, *Muribaculum*, *Akkermansia* and *UBA1819* were negatively correlated with subcutaneous fat, epididymal fat, body weight, FBG, serum TC, HDL-C, LDL-C and LPS. *Ruminococcaceae* *_UCG-014* and *UBA1819* belong to the family *Ruminococcaceae*. Previous studies have shown that *Ruminococcaceae* can produce SCFAs and maintain a healthy gastrointestinal tract (McNabney and Henagan, 2017; Videvall et al., 2020). In addition, *Ruminococcaceae* is reduced in older people and aged monkeys (Biagi et al., 2016; Duan et al., 2019). Therefore, we conclude that PDX treatment can modulate the gut microbiota in obese mice and significantly increase several beneficial microbes, including *Bacteroides*, *Parabacteroides*, *Alloprevotella*, *Muribaculum*, *Akkermansia*, *Ruminococcaceae* *_UCG-014*, and *UBA 1819*.

HFD increased circulating LPS, a component of gram-negative bacterial cell walls, which is in agreement with previous studies (Reilly et al., 2021; You et al., 2021). HFD can alter gut microbiota and intestinal wall permeability, and then LPS is transported via lymph to the circulation by incorporation into chylomicrons (CMs) (Hersoug et al., 2016). LPS can recognize toll-like receptor 4 (TLR4) and then activate NF-κB signaling, which promotes the secretion of MCP1 and recruitment of proinflammatory macrophages (Catrysse and van Loo, 2017). Furthermore, obesity leads to release of pro-inflammatory mediators such as IL-1β, IL-6 and MCP-1, and then causes circulating monocytes recruitment and macrophages accumulation in adipose tissue (Russo et al., 2021). Our results showed that PDX treatment reduced serum LPS and IL6 levels and significantly inhibited NF-κB signaling in the epididymal adipose tissue of HFD-fed mice. Accordingly, macrophage infiltration was alleviated in epididymal adipose tissue. Macrophages are generally classified into the M1 (expressing high levels of CD11c, TNF-α, IL-1β, IL-6, and Nos2) and M2 (expressing high levels of CD206, CD163, IL-10, IL-4, Arg1, Fizz1, Mrc1, and PPARγ) phenotypes, which represent proinflammatory and anti-inflammatory macrophages, respectively (Shapouri-Moghaddam et al., 2018). M1 macrophages can promote glycolysis to produce lactate instead of metabolizing pyruvate to acetyl-CoA, and M2 macrophages favor beta-oxidation of fatty acids and oxidative phosphorylation to produce energy-rich molecules, which are beneficial to tissue repair and anti-inflammation (Russo et al., 2021). We found that HFD increased infiltration of M1 macrophages and reduced M2 macrophages. In addition, PDX reduced M1 macrophage infiltration and resulted in macrophage polarization toward the M2 phenotype in the epididymal adipose tissue of HFD-fed mice. Moreover, PDX treatment reduced serum lipid profiles including TC, LDL-C and HDL-C levels and improved glucose tolerance and insulin sensitivity. Interestingly, lipogenesis-related genes such as Fasn and DGAT2 were downregulated in Part 1 mice with PDX supplementation, and lipolysis-related genes such as HSL, LPL and CD36 were upregulated in Part 2 mice with PDX treatment. The presumed reason may be that mice in the Part 1 experiment were lean, whereas the mice in the Part 2 experiment became significantly obese with metabolic disorders when the PDX administration began. Therefore, we conclude that PDX significantly alleviates adipose tissue inflammation in HFD-fed mice, which may be mediated by modulating the structure of the gut microbiota.

The lack of direct proof of the causal relationship between the gut microbiota and adipose tissue inflammation and glucolipid metabolism is the major limitation of the study. Future studies, including antibiotic treatment, FMT studies and oral treatment of live beneficial microbe, are needed, and we will continue our efforts to improve PDX studies and make clinical translation.

## Conclusion

In conclusion, our results indicate that PDX plays an important role in the prevention and treatment of obesity. PDX can improve glucolipid metabolism and adipose tissue inflammation in HFD-fed mice, which may be mediated by modulating the structure of the gut microbiota. PDX treatment promotes the growth of beneficial microbes including *Bacteroides*, *Parabacteroides*, *Alloprevotella*, *Muribaculum*, *Akkermansia*, *Ruminococcaceae* *_UCG-014* and *UBA1819* that are associated with obesity improvement. Therefore, we conclude that PDX may become a promising nondrug therapy to prevent and treat obesity.

## Data Availability

The raw data supporting the conclusion of this article will be made available by the authors, without undue reservation.
